# Parents’ Perceived Similarity to Their Children, and Parents’ Perspective Taking Efforts: Associations of Cross-Informant Discrepancies with Adolescent Problem Behavior

**DOI:** 10.3389/fpsyg.2016.00367

**Published:** 2016-03-15

**Authors:** Marc Vierhaus, Jana E. Rueth, Arnold Lohaus

**Affiliations:** Department of Psychology, Bielefeld UniversityBielefeld, Germany

**Keywords:** perceived similarity, adolescence, problem behavior, cross-informant reports, cross-informant discrepancies

## Abstract

The main goal of this study is to provide empirical evidence for a theoretical mechanism underlying cross-informant discrepancies (CID), which occur between reports of different informants (e.g., children/adolescents and parents) of children’s/adolescents’ problem behavior. Studies comprehensively corroborate the existence of CID. However, an explanation of CID is rudimentary and inconsistent. Respective research often suffers from methodological problems and is often atheoretical. Addressing these critics, this study uses polynomial regression and is based on research on mind perception and anchoring-and-adjustment theory. It was assumed that higher CID are associated with parents’ perceived similarity to their children, whereas lower CID are related to parents’ perspective-taking efforts. Analyses were based on *N* = 168 parent–child dyads (children’s mean age: 12.50 years). Reports on problem behavior displayed substantial mean differences and medium-sized correlations. Polynomial regressions on CID partly supported the influence of parents’ perceived similarity and perspective taking efforts on CID. Results are discussed in the context of a possible theoretical fundament for CID.

## Introduction

Cross-informant discrepancies (CID) refer to differences between reports of different informants on the same topic, in this case, adolescents’ emotional or behavioral problems. CID can exist between reports of different *external* informants (e.g., proxy reports of mother, father, or teacher) as well as between external informants and *internal* informants (self-reports of the adolescents themselves). Since Achenbach et al. (1987) published their meta-analysis (with almost 4,000 citations) based on 119 studies, a large amount of empirical research consistently has documented the occurrence of this phenomenon in clinical and community samples (e.g., Verhulst and van der Ende, 1992; Stanger and Lewis, 1993; Duhig et al., 2000; Achenbach et al., 2002; Waters et al., 2003; Vierhaus and Lohaus, 2008). This comprehensive research reliably has shown two main results for CID. First, relations between reports of different informants are of medium size, and they are lowest between adolescents’ self-reports and proxy reports and highest between two proxy reports (e.g., mother and father). Second, the mean of adolescents’ self-reported emotional or behavioral problems is higher than the mean based on proxy reports. However, the direction of these mean differences inverses in studies that focus on clinical instead of community samples.

Several studies published during the last three decades have evaluated factors and mechanisms that may explain, or at least be related to, CID. In this regard, studies evaluated characteristics of the child (e.g., sex, age, ethnical background, or problem type), parents (e.g., depression or parenting stress), or the family (e.g., marital status, lack of partner, or number of siblings). Summarizing the results of these studies, de los Reyes and Kazdin (2005) concluded that the relations of these characteristics to CID are inconsistent.

### Methodological Shortcomings in Research on CID

To a certain degree, the inconsistency of this pattern may be due to different methodological approaches used to measure and analyze CID across studies. Previous studies relied on a very direct way of estimating CID by difference scores, that is to say, by more or less subtracting individual scores of one source/informant (e.g., adolescents) from those of another (e.g., parent). Some of these studies *z*-standardized the score of each informant before subtracting, whereas a few other studies relied on a residual difference score. In the latter case, a first informant’s score was used to predict a second informant’s score, and CID equated to the difference between the predicted and the actual rating of the second informant. Finally, CID can be estimated based on a Latent Congruence-Model (LCM; Cheung, 2009). As a structural equation modeling approach, LCM provides the benefits of testing on measurement equivalence and of reduced random measurement errors.

However, these methodological approaches are based on a calculation of differences and have therefore been criticized extensively (de los Reyes and Kazdin, 2004; Edwards, 2009; Laird and De Los Reyes, 2013). First, difference scores bring up interpretational difficulties. Usually, adolescents report a higher mean of problem behavior than do parents in community samples, whereas in clinical samples, adolescents report a lower mean of problem behavior than do parents. However, there are substantial inter-individual variations regarding the direction of differences within each type of sample. Based on a community sample, Barker et al. (2007) could show that the proportion of adolescents reporting less problem behavior than mothers was nearly one third (27% regarding internalizing and 30% regarding externalizing problems). Thus, the interpretation of a relation between a difference score and another variable in terms of increased or decreased CID is oversimplified and misleading. Second, difference scores bring up mathematical difficulties and are related to their validity (extensively discussed by Laird and De Los Reyes, 2013). For one thing, the difference score cannot be distinguished from the two original scores used to create it. Mathematically, a correlation between a difference score and an external variable assumed to be related to it is determined by the variances of the original scores and the covariances between the original scores and the external variable. Otherwise, the relation of a difference score and an external variable imposes constraints to the relation of each original score to the external variable. Mathematically, these relation coefficients are constrained to be equal in magnitude and opposite in sign.

The current study adopts the suggestion made by de los Reyes and Kazdin (2004) to apply appropriate analytic strategies to address hypotheses related to CID directly. As outlined in the Method section, we used a polynomial regression approach (Edwards, 1994; Laird and LaFleur, 2014) to analyze whether the relation between adolescents’ self-reports and parents’ proxy reports is moderated by an external variable. The latter authors provide evidence that interaction terms in polynomial regression analyses, instead of various kinds of difference scores, represent variations of informant discordance and congruence, and therefore allow a direct test of hypotheses regarding CID. The usage of this approach in the current study and the interpretation of significant equation terms are described in the Method section.

### Theoretical Shortcomings of Research on CID

Beyond methodological differences, de los Reyes and Kazdin (2005) particularly criticized that research on CID lacks a theoretical foundation as a basis for research concepts. In this regard, De Los Reyes et al. (2013) theoretically defined and empirically underpinned *diverging operations* as a concept to delineate circumstances under which CID reflect meaningful information. Diverging operations are most assumable if the expression or impression of the behavior of interest (e.g., problem behavior) systematically varies across an external factor. On the one hand, this factor may be the *contextual variation or expression* of a behavior of interest. In this regard, Kraemer et al. (2003) have provided evidence that parents and teachers may provide discrepant reports because of the different settings (parents at home and teachers at school) in which they can observe the behavior of the child. On the other hand, systematic *social and cognitive mental processes* may account for the occurrence of discrepant reports because these processes may lead to different perceptions of external and internal informants (parents and children) of the same behavior. The current study addresses the recommendation of de los Reyes and Kazdin (2005) that future studies should contribute to a fundamental theoretical background of the emergence of CID. The theoretical background adopted in this study refers to a cognitive process (anchoring-and-adjustment, egocentric bias), which may influence the perspective of external informants (e.g., parents) and could therefore be linked to CID.

The ability to infer mental states of other people (e.g., attitudes, beliefs, goals, desires, intentions, or emotions) is called mind perception (Epley and Waytz, 2009). It is activated especially if the behavior of others needs to be explained, understood, or anticipated (Epley et al., 2008). In the literature, two mechanisms—*simulation* and *inference*—are postulated to underlie mind perception. Theory on *simulation* postulates that individuals use their knowledge regarding their own thoughts, intentions, emotions, and behavior to simulate the mind of another person, and by this, the means to anticipate thoughts, intentions, emotions, and future behavior of another person (Alicke et al., 2005). *Inference*, alternatively, is related to a developmental perspective: in the course of cognitive development, individuals build up a subjective theory of how the mind works and how it influences thoughts, intentions, emotions, and behavior. Based on this subjective theory, individuals infer the thoughts, intentions, emotions, and behavior of other individuals. To put it more clearly, inference and simulation focus on different processes, which happen to be a subjective theory in the case of inference, and self-related knowledge of the observer in the case of simulation.

Simulation and inference both have been incorporated within the concept of *anchoring and adjustment* (Epley et al., 2004), which suggests a successive dual-process model. It postulates that in order to judge and report on another person’s mind, feelings, or behavior, individuals initiate a simulation process—a rather automatic *anchoring*—which may be followed by an *adjustment* process focusing more strongly on inference and requiring additional cognitive resources. Simulation may lead to suitable results, for example, if the observer’s personality and situation do not differ much from those of the observed person. However, simulation may also be affected by an egocentric bias, which may increase the perceived difference between a person’s own perspective and that of the other person if this other person is assumed to be similar, but is indeed different.

Because a correction of an automatic judgment based on simulation requires additional cognitive resources, it is moderated by several variables. Besides time pressure and motivation (Epley et al., 2004), *perceived similarity* is assumed to influence the anchoring-and-adjustment process. Using functional neuroimaging, Mitchell et al. (2006) were able to show that self-referential mentalizing activates the same region of the ventral medial prefrontal cortex as does mentalizing about a person perceived to be similar. By contrast, mentalizing about a dissimilar person activates a more dorsal subregion of the medial prefrontal cortex. The overlap of activation between judgments of oneself and a similar other (see also Ames et al., 2008) underlines that reports on characteristics of similar others may be the result of an automatic simulation process based on the informant’s knowledge regarding the informant’s own thoughts, intentions, emotions, and behavior. Kenny (1994) assumed that the self may serve as a basis for the perception of other family members so that, for example, the perceiver’s own problem behavior may be used as a basis for rating the problem behavior of other family members. Although Kenny (1994) labels this process assimilation, he seems to assume similarity to be the underlying mechanism. Manders et al. (2009) analyzed the reports of each family member on each other family member’s problem behavior. Based on the social relations model (SRM; Cook and Kenny, 2004), the results reported by Manders et al. (2009) have shown that within-family perceptions of problem behavior are substantially influenced by a perceiver effect. This means that a family member (perceiver) rates the problem behavior of the other family members (targets) very similarly. Thus, although it seems assumable that family members are well-acquainted with each other such that they are able to provide differential reports on other family members, this result may reflect a within-family assimilation process for which the self is likely to serve as a basis.

### Hypotheses

Besides hypotheses that aim to replicate fundamental results on CID documented in the research field (Hypotheses 1 and 2), we have formulated two additional hypotheses (Hypotheses 3 and 4), which are based on the theoretical and empirical background regarding mind perception. As a replication of former results, we hypothesized that adolescents’ self-reports on problem behavior and parents’ proxy reports on problem behavior are positively related (Hypothesis 1), and that adolescents’ self-reports on problem behavior exceed parents’ proxy reports on problem behavior (Hypothesis 2). With reference to the anchoring-and-adjustment theory, we hypothesized that increased perceived similarity is related to increased discrepancies between the parents’ reports and the reports of their children (Hypothesis 3). Thus, if parents base their judgments mainly on self-related knowledge (*simulation*) affected by egocentric bias, it is more likely that parents and their children will arrive at divergent judgments. In contrast to the former effect, we assumed that decreased discrepancies are related to increased *perspective-taking efforts* (Hypothesis 4) because perspective-taking efforts can be regarded as additional cognitive resources promoting *inference*. We tested all hypotheses regarding two broad domains of problem behavior (i.e., internalizing and externalizing behavior) to evaluate a possible domain specificity of the assumed relations. For example, regarding the effect of perspective-taking efforts, we assumed that this effect may be more pronounced for internalizing behavior because it may be less observable to external informants.

## Materials and Methods

### Sample and Procedure

Analyses were based on a sample of *n* = 168 German mother–child dyads with 60% girls and 40% boys. The adolescent children’s mean age was *M* = 12.50 years (*SD* = 1.71, range: 10–16 years). Originally, *N* = 223 adolescents were contacted via headmasters of four schools. The adolescents’ participation in the study was on a voluntary basis and required their parents’ permission. Of the original sample of contacted adolescents, 17.9% had no permission to participate in the study. The remaining 183 adolescents completed the questionnaires during school classes, supported by one of three trained graduate students in case they had problems understanding the items. Participants needed about 20–25 min to fill out the questionnaire. Afterwards, the adolescents were provided with an enveloped questionnaire addressed to their main caregiver. The completed and sealed parent questionnaires had to be returned to the school. A total of 174 (95.1%) of the adolescents returned this questionnaire, of which six were completed by fathers. Because of this unbalance, the analyses were based on the remaining *n* = 168 mother–child dyads (75.3% of the original sample of contacted adolescents). All adolescents were Caucasians from lower to upper middle-class socioeconomic backgrounds. The recruitment of the samples and the study’s procedure were in accordance with the ethical guidelines of the American Psychological Association (APA) and the Society for Research in Child Development (SRCD). This means that participation of the children required their parents’ permission. The children and their parents participated on a voluntary basis and were completely informed about the details and goals of the study. The trained students were mindful of preserving children’s anonymity while they completed their questionnaires. The study was approved by an independent ethical review board.

### Measures

#### Adolescent Problem Behavior

The four problem scales of the self-rated and the informant-rated German version of the Strengths and Difficulties Questionnaire (SDQ; Klasen et al., 2000) were used to assess self-reports of children/adolescents and parental reports on their children’s/adolescents’ problem behavior. The four scales are widely used to assess (a) emotional symptoms (e.g., “I worry a lot”; “My child has many worries, often seems worried”), (b) peer relationship problems (e.g., “I am usually on my own”; “My child is rather solitary, tends to play alone”), (c) conduct problems (e.g., “I fight a lot”; “My child often fights with other children or bullies them”), and (d) hyperactivity (e.g., “I am easily distracted”; “My child is easily distracted, its concentration wanders”). Each of the four scales consisted of five items. As proposed by Goodman et al. (2010) for low-risk samples, the broadband scales of internalizing and externalizing problem behavior were computed by averaging the items of the scales *emotional symptoms* and *peer relationship problems* (forming the internalizing scale), as well as of the items of the scales *conduct problems* and *hyperactivity* (forming the externalizing scale). The internal consistencies of adolescents’ self-reports were α = 0.65 regarding internalizing and α = 0.62 regarding externalizing problem behavior. The respective values for parents’ proxy reports were α = 0.67 and α = 0.71. The internal consistencies are largely in line with those reported by Goodman et al. (2010), based on the English version of the SDQ.

#### Perceived Similarity

Parents’ extent of ability to perceive aspects of their own personality as similar to those of the personality of their child was assessed by the scale *Similarity* of the Family Diagnostic Test System (FDTS; Schneewind et al., 1985). The scale consists of nine items (e.g., “My child feels, thinks and behaves just like me”) on a four-point rating scale. The internal consistency of α = 0.95 reported by Schneewind et al. (1985) could be largely replicated in the present study (α = 0.88). Ranging from 1 to 4, the mean of the scale was *M* = 2.90 (*SD* = 0.58).

*Perspective-taking efforts* were assessed by the scale “Taking the perspective of the child” of the Scales for the Measurement of Supportive Parental Behavior (Peterander, 1993). This scale contains nine items (e.g., “I often try to see situations from the point of view of my child”; “I think about how my child gets along with certain experiences”) on a four-point rating scale, and displays a very good internal consistency of α = 0.85, which is similar to the one reported by Peterander (1993). Ranging from 1 to 4, the mean of the scale was *M* = 3.03 (*SD* = 0.60).

### Statistical Analyses

All analyses were conducted using the statistical software R (Version 3.1.2). Regarding Hypotheses 1 and 2, *t*-tests (paired) and Pearson correlations were computed. Regarding Hypotheses 3 and 4, polynomial regressions with interaction terms were conducted as proposed by Edwards (1994) and by Laird and LaFleur (2014). Perceived similarity and perspective-taking efforts were treated as moderators of the relation between adolescents’ self-reports and mothers’ proxy reports on internalizing and externalizing problem behavior in four separate hierarchical regression analyses. Each regression model included adolescents’ self-report on problem behavior (either internalizing or externalizing), mothers’ report on the moderator (either perceived similarity or perspective-taking efforts), and the respective interaction term as predictors, as well as mothers’ proxy reports on problem behavior (either internalizing or externalizing) as criterion. The significance of an interaction term indicated that the relation between self-reports and proxy reports on problem behavior is increased (plus-signed interaction) or decreased (minus-signed interaction) by the respective moderator. As recommended by Edwards (1994) and in line with the approach of Laird and LaFleur (2014), to meet the potential complexity of relations, each regression model additionally included quadratic terms (adolescents’ report squared, moderator squared) and related interaction terms (adolescents’ report × moderator squared, adolescents’ report squared × moderator). As four polynomial regressions were conducted, the significance level was adjusted to α = 0.013 (0.05/4). With respect to Hypotheses 3 and 4, all four directional hypotheses were one-sided tested.

## Results

The first part of this section provides results regarding the correlations (Hypothesis 1) and mean differences (Hypothesis 2) between adolescents’ self-reports and parents’ proxy reports on internalizing and externalizing problem behavior. The second part of the result section is concerned with assumptions based on anchoring and adjustment theory and reports on the relation between CID and perceived similarity (Hypothesis 3), as well as between CID and perspective-taking efforts (Hypothesis 4).

### Cross-Informant Reports: Relations and Mean Differences

Correlations and mean differences between self-reports of children/adolescents and the proxy reports of their parents were analyzed (**Table 1**).

**Table 1:** Means and standard deviations as well as correlations of and mean differences between self-reports and proxy reports on adolescent problem behavior.

	Mothers’ report	Adolescents’ report	Mean difference between parent and adolescent Report			Correlation between parent and adolescent report	
	*M (SD)*	*M (SD)*	*df*	*T*	*p*	*r*	*p*
Internalizing	1.36 (0.28)	1.54 (0.31)	167	6.53	<0.001	0.32	<0.001
Externalizing	1.39 (0.28)	1.56 (0.26)	167	6.24	<0.001	0.20	0.008

As can be seen from **Table 1**, with respect to internalizing and externalizing problem behavior, children’s/adolescents’ self-reports display a significantly higher mean than do parents’ proxy reports. Additionally, the correlations between both reports are significant regarding internalizing and externalizing behavior. Thus, the results confirm Hypotheses 1 and 2.

### Cross-Informant Discrepancies: Relations to Perceived Similarity and Perspective-Taking Efforts

Hypotheses 3 and 4 assume that the relation between self-reports and proxy reports is moderated by perceived similarity and perspective-taking efforts, respectively. Each of these hypotheses was tested by two polynomial regressions using the self-reports/proxy reports on internalizing or externalizing problems as predictor/criterion and either perceived similarity or perspective-taking efforts as a moderator. The results of the four regressions are summarized in **Table 2**.

**Table 2:** Polynomial regression analyses predicting mothers’ reports on problem behavior from adolescents’ self-reports on problem behavior and mothers’ reports on perceived similarity and perspective taking efforts.

	Mothers’ report on internalizing behavior		Mothers’ report on externalizing behavior	
Moderator	Perceived similarity	Perspective taking	Perceived similarity	Perspective taking
Predictor	β (p)	β (p)	β (p)	β (p)
SR-PB	0.17 (0.06)	0.21 (0.02)	0.22 (0.01)	0.15 (0.08)
SR-PB _Squared_	0.14 (0.06)	0.06 (0.23)	-0.10 (0.11)	-0.07 (0.19)
Moderator	-0.15 (0.13)	0.05 (0.29)	-0.20 (0.02)	0.18 (0.03)
Moderator _Squared_	0.12 (0.08)	0.02 (0.39)	0.01 (0.47)	0.07 (0.20)
SR-PB × Moderator	0.05 (0.27)	-0.15 (0.05)	-0.20 (0.01)	-0.05 (0.27)
SR-PB _Squared_ × Moderator	0.06 (0.28)	0.30 (0.00)	0.12 (0.12)	-0.11 (0.11)
SR-PB × Moderator _Squared_	0.15 (0.08)	0.13 (0.12)	0.01 (0.47)	0.13 (0.11)
*R*^2^	0.15	0.19	0.11	0.08

*SR-PB, Self-reported problem behavior.*

Perceived similarity did not moderate the relation between reports on internalizing behavior (β = 0.05, *p* = 0.271). In addition, there were no significant quadratic interaction terms (β = 0.06, *p* = 0.280; β = 0.15, *p* = 0.075). By contrast, the model including externalizing behavior as predictor/criterion showed a significant linear interaction term (β = -0.20, *p* = 0.013).

**FIGURE 1:**
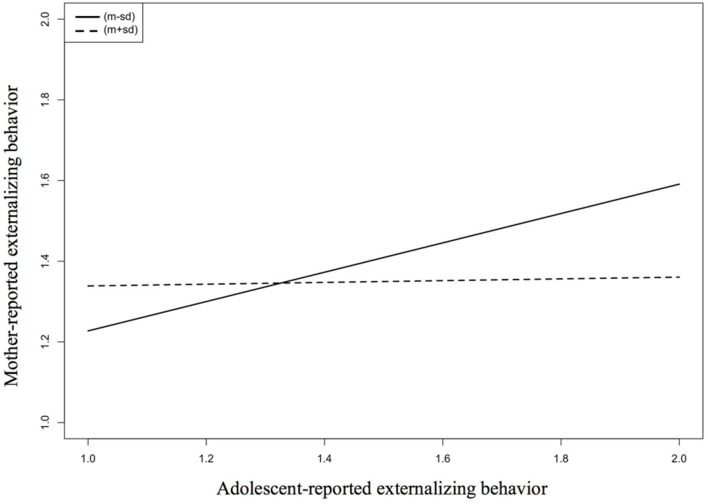
**Predicting mother-reported externalizing behavior as a function of adolescent-reported externalizing behavior and perceived similarity (moderator)**.

As indicated by the minus-signed interaction, the discrepancy between adolescents’ self-reports and mothers’ proxy reports on externalizing problem behavior is lower if perceived similarity is high (dashed line in **Figure 1**). Partly verifying Hypothesis 3, this indicates that CID for externalizing behavior (but not for internalizing behavior) is increased for high levels of perceived similarity.

Perspective-taking efforts did not moderate the relation between reports on internalizing behavior on a linear level (β = -0.15, *p* = 0.052). However, the consideration of quadratic terms showed a significant effect (β = 0.30, *p* = 0.003). By contrast, the model including externalizing behavior as predictor/criterion indicated no significant interaction term.

**FIGURE 2:**
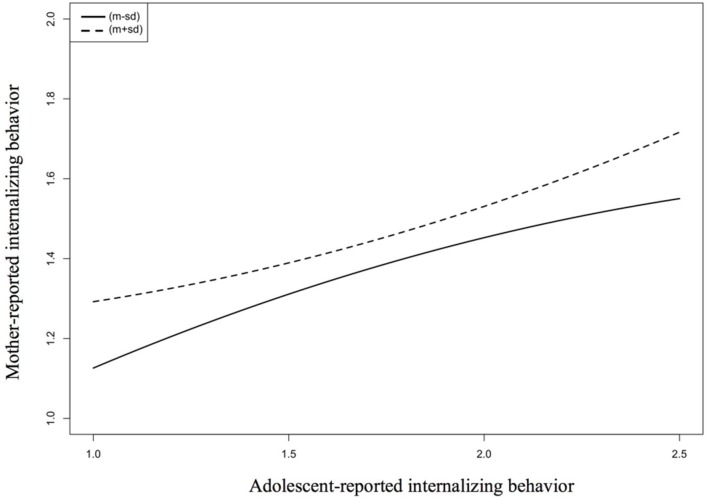
**Predicting mother-reported internalizing behavior as a function of adolescent-reported internalizing behavior and perspective-taking efforts (moderator)**.

As shown in **Figure 2**, the quadratic relation between adolescents’ reports and mothers’ reports on internalizing behavior is positive (accelerated) at high levels of mothers’ perspective-taking efforts, but is negative (decelerated) at low levels of mothers’ perspective-taking efforts. Thus, partly verifying Hypothesis 4, this indicates that low levels of CID are associated with high levels of perspective-taking efforts (but only for internalizing, not for externalizing behavior).

## Discussion

Significant and systematic differences regarding means of and relations between self-reports of children or adolescents and their parents’ proxy reports on problem behavior (Cross-Informant Discrepancies; CID) are well documented in the research literature (de los Reyes and Kazdin, 2005). Two fundamental results could be replicated by the current study. First, the correlation between self-reports and proxy reports on internalizing and externalizing behavior is positive (Hypothesis 1), but medium-sized at best. Second, as the results are based on a community sample of parent–child dyads, it was assumed in Hypothesis 2 (and corroborated by the results) that the mean of internalizing and externalizing behavior is significantly higher in children’s/adolescents’ self-reports than in parents’ proxy reports. Both results coincide with other studies and underline the replicability and prominence of CID.

In contrast to the empirical corroboration of the occurrence of CID (in community as well as in clinical samples), the relations to potentially influential factors are often inconsistent across corresponding studies. Beyond methodological differences (especially regarding mathematical limitations and interpretability of difference scores), de los Reyes and Kazdin (2005) particularly criticized that research on CID is lacking a theoretical framework. This study focuses on the anchoring-and-adjustment theory, which assumes that the judgment of an external informant on characteristics of a target person involves two steps: initially and rather automatically, an informant *anchors* using a cognitive simulation based on self-related knowledge, and subsequently *adjusts* this judgment.

Based on this construct, it was hypothesized that perceived similarity is related to increased CID (Hypothesis 3) because the more parents perceive themselves to be similar to their children, the more their self will serve as a basis to assess their children (egocentric bias). The results show that the discrepancy of parent-reported externalizing behavior and adolescent-reported externalizing behavior is associated with high levels of perceived similarity. However, regarding internalizing behavior, perceived similarity did not show a moderation effect. Thus, the results partly corroborate Hypothesis 3 and coincide with the results of Kenny (1994) and Manders et al. (2009). It seems reasonable that reports of parents are not interchangeable with children’s self-reports, and that they may provide unique information (Achenbach et al., 2005) regarding the emotional and behavioral problems of their children. However, in light of the relation between perceived similarity and CID, this conclusion should be drawn carefully because of a possible egocentric bias.

The question that follows is whether and how this effect can be reduced or counteracted. One possibility is to account for parents’ perspective-taking efforts. In line with Hypothesis 4, the results show that high degrees of perspective-taking are related to an increased congruence between adolescent-reported and parent-reported internalizing behavior. Based on the duality of the anchoring-and-adjustment model, this result indicates that parents’ perspective-taking efforts reflect an adjustment process, which follows the initial anchoring process. It appears that CID (regarding internalizing but not regarding externalizing behavior) are reduced if parents invest additional cognitive resources (deliberate and effortful attention, intentional control) to adjust a rather automatically formed egocentric anchor.

Although the results of the moderation analyses support the two central hypotheses of the study, it is important to note that support depends on the type of problem behavior: whereas the relation of CID and perceived similarity (Hypothesis 3) could be demonstrated regarding externalizing behavior, the relation of CID and perspective-taking efforts (Hypothesis 4) finds support regarding internalizing behavior. This specificity has to be interpreted in terms of anchoring-and-adjustment theory. Regarding externalizing behavior, the parents’ focus seems to be on similarities to the child, and there seems to be no influence of perspective taking. Regarding children’s internalizing problem behavior, the cognitive focus of parents (perceived similarity) does not seem to influence the adjustment process. However, additional cognitive efforts (perspective-taking) seem to counteract a possible egocentric bias. Thus, based on the specificity of the results, the main conclusion is that for different types of problem behavior, different variables may affect the judgment process.

It is widely recommended that the assessment of emotional and behavioral problems of children and adolescents should be based on different informants (Kraemer et al., 2003) because the perspective of each informant provides unique, non-interchangeable information (e.g., Vierhaus and Lohaus, 2008). However, the aggregation of cross-informant information complicates the diagnostic process as well as the planning and initiation of interventions if the source of CID remains unexplained. Based on theoretical considerations, the present study sheds light on processes that may underlie CIDs. Overall, the results support the assumption that the degree to which parents perceive their children as similar to themselves seems to be related to CID. Conversely, perspective-taking efforts seem to counteract cognitive processes that may be used to decrease CID.

The results of the study point to the fact that mental processes may play an important role by influencing the proxy reports of parents and in turn the relation of their reports to the self-reports of their children. One of these processes – Inference or Theory of Mind (TOM) – has been empirically discussed with respect to its development during childhood. Therefore, a developmental perspective on the relatedness of TOM and CID (and its influence on children’s self-reports) appears obvious. Indeed, the concept of TOM does not only refer to the capacity to mentalize (attribute mental states to others) but also to the capability of remembering events and to introspection (Perner et al., 2007). Both, remembering events and introspection are relevant aspects of providing self-reports (e.g., children and adolescents are asked to report on emotional problems during the last 6 months). However, as outlined by de los Reyes and Kazdin (2005) the effect of children’s age on CID is very inconsistent (with studies reporting a significant positive or negative or even no significant relation). In addition, studies on TOM indeed provide evidence that the capability of introspection may develop later than mentalizing (6–8 vs. 4–5 years, respectively). Children in studies on CID are about 11–12 years of age on average and therefore much older. Thus, both capabilities should be available. However, there may be interindividual differences regarding TOM even in older children and adolescents and future studies may focus on the relation between TOM and CID representing a possible influence on children’s self-reports.

Even though the present study provides unique contributions to the field, some critical points should be mentioned. First, it should be noted that mothers completed more than 95% of the parent questionnaires. Therefore, the generalizability is restricted, and whether the same mechanisms underlie fathers’ judgment process and whether they are related to CID in the same way needs further investigation. Second, it is worth mentioning that the applied methodological approach has rarely been used in other studies on CID before (it was suggested by Laird and LaFleur, 2014). In addition, second-order scales instead of the conventional first-order problem-scales of the SDQ were used. Although suggested by Goodman et al. (2010) for subclinical samples, this procedure has rarely been used before. It should also be noted that the internal consistencies of the second-order scales of the German version were largely in line with those reported by Goodman et al. (2010), although they were consistently lower. As a consequence, results of the current study can only conditionally be compared with results of other studies in the research field. However, as noted by de los Reyes and Kazdin (2004), current and frequently used ways of measuring/estimating CID are associated with several problems. It is an additional challenge for future studies to rely on methods that estimate CID in the most appropriate way, and the approach used in the current study may represent an approximation to this demand.

## Author Contributions

MV: concept of the study, statistical analyses, writing of the article. JR: recruitment of the sample, management of data collection, additional writing, proof reading. AL: additional writing, proof reading.

## Conflict of Interest Statement

The authors declare that the research was conducted in the absence of any commercial or financial relationships that could be construed as a potential conflict of interest.
